# D‐Alloantibody Titration Assessment Study: In Search of a Common Antibody Titration Platform—A BEST Collaborative Study

**DOI:** 10.1111/trf.70022

**Published:** 2025-12-12

**Authors:** Fatima A. Aldarweesh, Rim Abdallah, Ingrid Perez Alvarez, Jennifer Andrews, Therese M. Chlebeck, Jessica Clower, Aisling Costelloe, Dolores Figueroa, Chloe George, Michael Evans, Sarah Ilstrup, Ellen B. Klapper, Angela Mueller, Hannah Peterson, Terry Rees, Jina Seo, Arrey N. Takang, Claudia S. Cohn, the Biomedical Excellence for Safer Transfusion (BEST) Collaborative

**Affiliations:** ^1^ Department of Pathology University of Chicago Chicago Illinois USA; ^2^ Vitalant Scottsdale Arizona USA; ^3^ Cedars Sinai Los Angeles California USA; ^4^ Department of Pathology, Microbiology and Immunology Vanderbilt University Medical Center Nashville Tennessee USA; ^5^ Department of Laboratory Medicine and Pathology University of Minnesota Minneapolis Minnesota USA; ^6^ Irish Blood Transfusion Service Dublin Ireland; ^7^ Welsh Blood Service Pontyclun UK; ^8^ Intermountain Health Salt Lake City Utah USA; ^9^ M‐Health, Fairview Hospital Minneapolis Minnesota USA

**Keywords:** alloimmunization, antibody titer, D‐antigen, gel‐based testing, hemolytic disease of the fetus and newborn (HDFN), prenatal screening, Rh, tube testing

## Abstract

**Background:**

Alloimmunization against D‐antigen can cause severe Hemolytic Disease of the Fetus and Newborn (HDFN). Traditionally, anti‐D‐titers are measured using a saline indirect antiglobulin test (tube testing). Anti‐D‐titers ≥8 during pregnancy trigger an escalation in maternal care. Tube testing is labor‐intensive and known for imprecision. Automated gel‐based titration is more sensitive and precise than tube titration for the detection of anti‐D. A gel titer correlated with potential fetal anemia has not been established, as studies comparing gel and tube titers provide widely variable results. This multicenter study tested anti‐D samples in parallel to characterize the difference in sensitivity between tube and automated gel assays.

**Study Design and Methods:**

Patients alloimmunized to RhD had samples tested using tube and automated gel titration methods. A total of 647 samples were tested in parallel. A subset of 141 samples also had anti‐D levels quantified using continuous flow analysis (CFA). Controlled lots of R_2_R_2_ red blood cells and standardized reagents were utilized.

**Results:**

Results demonstrated that gel‐based methods yielded mean titers 2.5–3 dilutions higher than tube; this difference diminished at tube titers >128. Notably, several samples previously considered negative by tube were positive by gel. Anti‐D levels quantified by CFA demonstrated a good correlation with tube and gel testing (*R* = 0.75–0.9 for tube; *R* = 0.85–0.89 for gel).

**Discussion:**

A tube titer of 8 to 16 correlates with an automated gel titer of 32–128 when R_2_R_2_ cells are used. Results using the CFA method correlate well with tube and gel analyses.

**Abbreviations**
AHG: indirect antiglobulin
BEST: biomedical excellence for safer transfusion
CFA: continuous flow analysis
GA: gestational age
HDFN: hemolytic disease of the fetus and newborn
IRB: institutional review board
RhIg: Rh immunoglobulin

## 1 INTRODUCTION

Antibodies against the D‐antigen can cause a severe form of Hemolytic Disease of the Fetus and Newborn (HDFN), leading to potentially severe complications such as hyperbilirubinemia, kernicterus, and erythroblastosis fetalis. HDFN affects neonates worldwide, and despite advances in prophylaxis and management, it remains a significant cause of morbidity and mortality. While the incidence of HDFN has decreased in high‐income countries due to effective screening and prophylaxis, there are still pregnant patients with D‐antibodies who must be carefully monitored as elevated anti‐D‐titer levels are a marker for fetal anemia.^1^, ^2^, ^3^, ^4^

The titration of an alloantibody to a red cell antigen is a useful semiquantitative screening tool that can detect an increased production of maternal antibody during pregnancy. Traditionally, prenatal titers are measured using a saline indirect antiglobulin (anti‐human globulin, AHG) test, which is performed in a test tube (tube testing). When the anti‐D tube titer increases fourfold (2 dilution increase) or rises above an accepted threshold of 8–16, obstetricians consider this a critical value and often increase fetal monitoring for anemia.^5^, ^6^, ^7^

However, the value of tube testing is limited by its lack of precision, which is related to the quality of the dilutions performed, interobserver variability, different commercial reagent red blood cells (RBCs) that give quantifiably different results,^8^ and varying policies for interpreting results. This imprecision leads to inefficiency as laboratories often test historical samples alongside current samples for comparison. An automated gel technology system that fully prepares serial dilutions, pipets all sample/reagents, and reads and records test reactions provides a more reliable method for anti‐D‐titer testing with objective grading, stable reactions, and precise pipetting. Yet, the lack of standardized critical titer levels for gel‐based methods poses a challenge. Studies that have tried to correlate tube and gel testing have provided widely varying results, with gel testing shown to be anywhere from 1 to 8 dilutions more sensitive than tube testing,^9^, ^10^, ^11^ or 1–2 dilutions less sensitive than tube testing.^12^, ^13^ To address these discrepancies, a scoping review found that the lack of correlation between tube and gel was largely due to the red blood cell reagents used to perform the titer, with the inherent imprecision of tube testing a contributing factor.^14^, ^15^, ^16^

This study sought to compare the performance of tube and automated gel titration testing in patients with anti‐D alloimmunization and to evaluate the correlation between titer levels obtained using these two methods. A third method of quantifying D‐antibody levels, the continuous flow assay (CFA), was also compared to tube and gel testing. The CFA uses segmented flow analysis to quantify the anti‐D level in the sample by comparing it to a standard curve created with a known amount of anti‐D, facilitating determination of the sample's concentration.^17^ The CFA is used for initial detection of anti‐D and is then repeated at 4‐week intervals until 28 weeks gestational age (GA), and every 2 weeks thereafter.^18^

In this study, standardized methods were used across institutions to assess the correlation between these assays and find a gel titer for anti‐D that correlated well with the 8 to 16 anti‐D critical tube titer currently used by most obstetricians. To the best of our knowledge, this study is the most extensive standardized validation study comparing automated gel and tube titration results.

## 2 STUDY DESIGN AND METHODS

A total of eight sites contributed samples for this study. Six sites tested their local samples from adult patients with anti‐D alloimmunization using tube and gel technology. Two additional sites contributed samples from pregnant patients who had been tested using the CFA. These samples were frozen and shipped to one of the six sites for parallel testing with tube and gel methods. Samples were excluded if patients had received Rh immunoglobulin (RhIg) within the preceding 3 months. A total of 647 samples were tested; however, 12 undiluted (neat) samples with no agglutination seen in both tube and gel were removed from the final analysis, leaving a total of 635 paired samples for analysis. Each sample was tested on the same day under similar conditions, undergoing tube and gel testing while fresh or after a single freeze–thaw cycle. The resulting anti‐D‐titers were recorded in a REDCap (Vanderbilt University, Nashville, Tennessee, United States) registry, adhering to a standardized protocol. Serial doubling dilutions in saline (neat, 1:2, 1:4, 1:8, 1:16, 1:32, 1:64, 1:128, 1:256, 1:512, and 1:1024) were performed on all samples, with some sites extending dilutions to 1:2048. All sites followed the same study protocol for the comparison of each test method.

### 2.1 Testing methods

For the automated testing, each site utilized the Ortho Vision® Analyzer (QuidelOrtho Raritan, NJ, USA) in routine use in their laboratory. Different R_2_R_2_ donors were used to make the 3% and 0.8% cells. However, the same lot of R_2_R_2_ antibody detection cells were provided for tube tests (3% SELECTOGEN®, QuidelOrtho, Raritan, NJ, USA) and ID‐MTS Gel™ Test (0.8% SELECTOGEN® QuidelOrtho, Raritan, NJ, USA). Each site utilized its stock antiglobulin tube‐based test reagent for the tube titration testing and ID‐MTS Gel cards for the column agglutination test. Titers were read as the highest dilution to yield 1+ agglutination.

Weak agglutination was not considered positive. Titer values were expressed as the inverse of the dilution factor.

CFA quantified the D‐antibody level via interaction with a standardized D‐antigen.^18^ The interaction generates a signal, and the concentration of antigen is quantified based on the intensity of the signal.

Data collected for each specimen included site name, collection date, specimen thaw status, obstetric patient status, R_2_R_2_ reagent RBC cell lot number and expiration date, AHG lot number and expiration date, and the presence of any additional alloantibodies. All participating study sites obtained the necessary institutional review board (IRB) approvals.

### 2.2 Statistical analysis

A total of 12 samples with tube and gel titers reported as 0 were excluded from the analysis.

The remaining 635 paired tube and gel samples were evaluated for concordance. Relationships between gel and tube titers were examined using generalized additive models of log2 gel:tube ratio with a smooth term predictor for log2 gel titer or log2 tube titer (to allow for nonlinear relationships between the titer and the ratio) and factor terms for categorical predictors including fresh versus freeze/thaw, presence of additional red blood cell antibodies beyond anti‐D, and patient pregnant status. Results are summarized using estimated marginal means with 95% confidence intervals. Relationships between titers and CFA measurements were examined using similar models. Pearson correlations were also used to summarize relationships between continuous measures. Analyses were conducted using R version 4.4.2.

## 3 RESULTS

**FIGURE 1:**
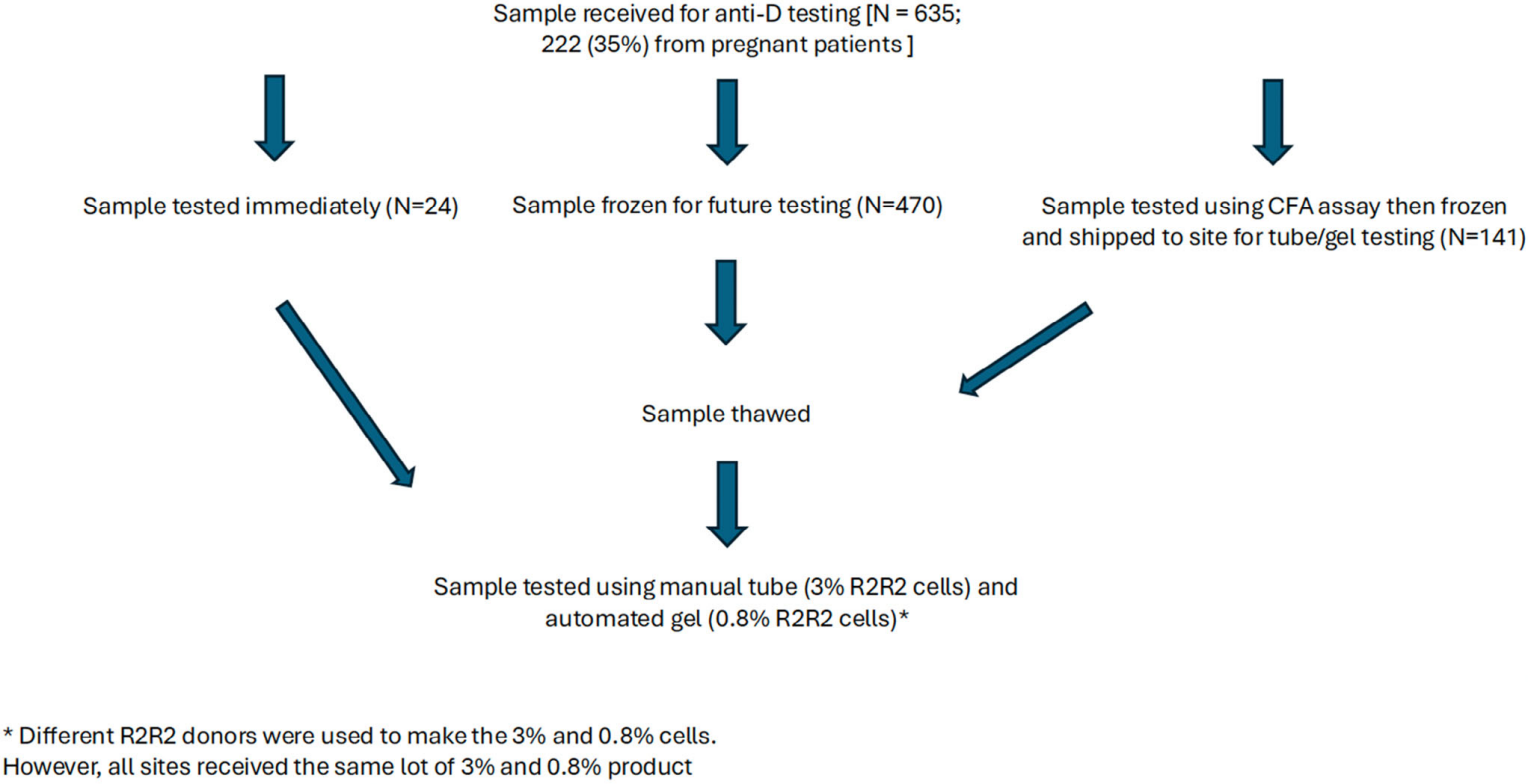
Diagram demonstrating the flow of samples in this study.

**FIGURE 2:**
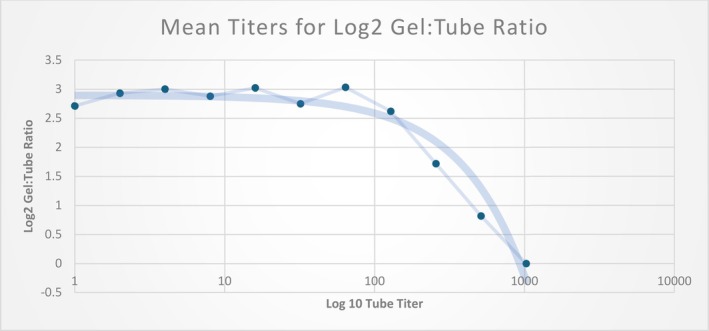
Tube titers versus gel to tube (log2) titer ratios.

**FIGURE 3:**
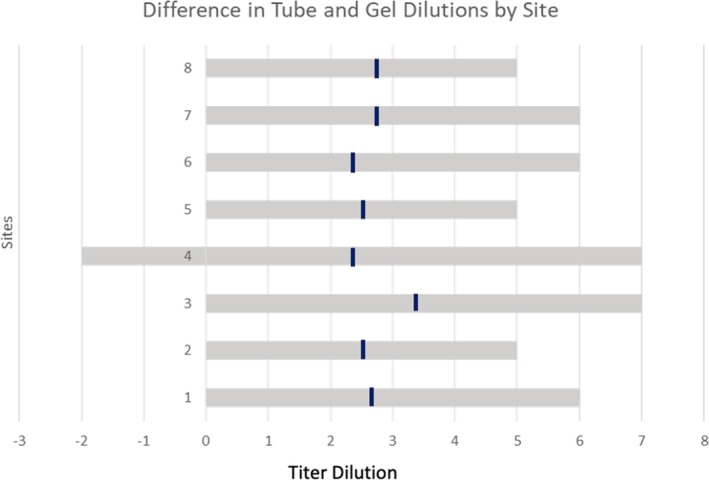
A comparison of tube to gel dilutions across the sites. The dilutional differences between tube and gel ranged from 4 to 7. The mean differences ranged from 2.30 dilutions to 3.37.

The study compared the sensitivity of anti‐D‐titration tests performed with tube and gel technologies on 635 samples obtained from eight sites. Figure 1 shows the overall flow for sample testing. Figure 2 shows that, overall, gel‐based methods produced mean titer results that were 2.5‐ to 3‐fold dilutions higher than tube‐based methods, with higher titer samples demonstrating a decrease in this delta as they approached the maximum titer of 1024. Specifically, a tube titer of 8 correlated with a gel titer in the range of 32 to 64. Similarly, a tube titer of 16 correlated to a gel titer within the range of 64 to 128. Table 1 provides greater detail, highlighting the fact that 155 samples that were negative by tube testing were positive by gel testing, with titers ranging from 1 to 64 (data not shown). Table 2 demonstrates the low level of interlaboratory variability. However, as shown in Figure 3, there is also a wide range in tube to gel correlation, with a 4–7 dilution difference when comparing tube and gel. As this range is consistent across sites, it most likely reflects the lack of precision associated with tube titration.

**TABLE 1:** Samples were tested in parallel using tube and gel technologies.

Tube Titer	Number of samples	Mean Log2 Gel:Tube ratio
0	155	2.44
1	49	2.71
2	58	2.93
4	52	3.00
8	60	2.88
16	45	3.02
32	57	2.75
64	40	3.03
128	47	2.62
256	32	1.72
512	11	0.82
1024	29	0.00

*Note*: The gel to tube titer ratio was transformed using log base 2. This transformation allows serial titers to be expressed in a linear manner, with 1–2 = 1 dilution, and 1–4 = 2 dilutions. The gel titer was 2–3 dilutions more sensitive than tube from dilutions 0–128, after which the difference in titer results between methods declined.

**TABLE 2:** The number of samples tested at participating sites with mean difference between tube and gel titers and range.

Site	Number of samples	Mean Log2 Gel:Tube ratio	Range in dilutions between tube and gel (min–max)
1	18	2.67	6 (0–6)
2	54	2.46	4 (1–5)
3	52	3.37	6 (1–7)
4	168	2.33	7 (−2–5)
5	52	2.56	4 (1–5)
6	150	2.3	6 (0–6)
7	98	2.72	6 (0–6)
8	43	2.74	5 (0–5)
Total	635		

*Note*: The dilutional difference between tube and gel ranged from 4 to 7.

### 3.1 CFA compared to tube and gel technologies

**FIGURE 4:**
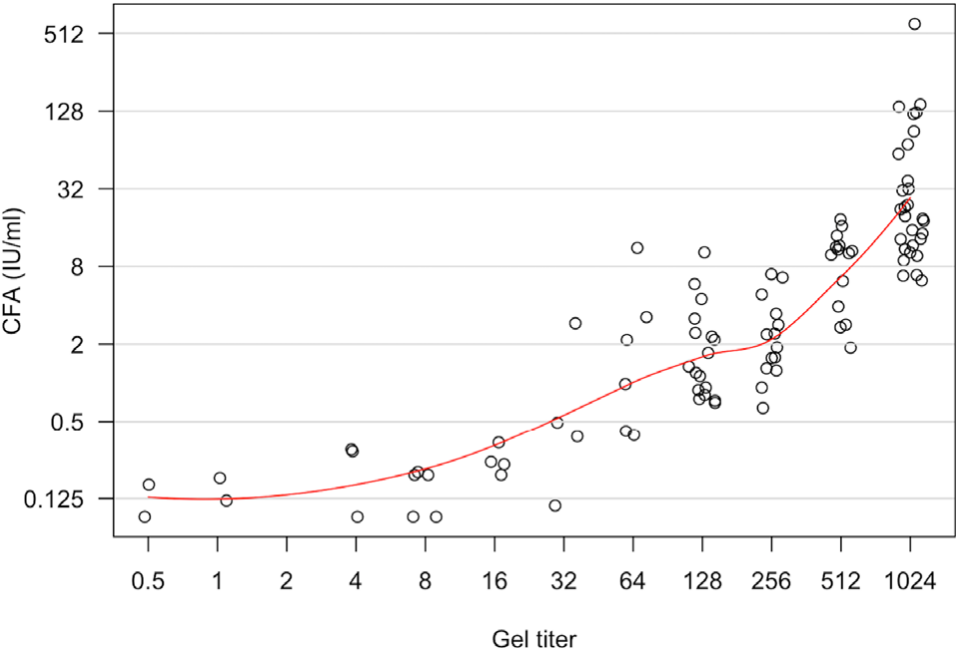
Gel titer assay (log2) versus Continuous Flow Assay (IU/ml) from one center.

There were 141 samples included in comparison testing from two sites (Sites 7 and 8), where the D‐antibody levels were first obtained using the CFA method. These samples were shipped frozen to a third site where they were tested in parallel using tube and gel. Figure 4 demonstrates the correlation between the CFA results versus tube and gel testing. Overall, gel testing showed a good correlation, with Pearson R values of 0.85 and 0.89 for sites 7 and 8, respectively. The R values calculated for CFA versus tube testing are 0.9 and 0.75 for these sites, respectively. The correlations at the low‐ and high‐end of the testing range were good. More specifically, a CFA ≤ 0.4 IU/mL, which is considered low risk for HDFN,^19^ was equivalent to a mean titer of 2 and 16 by tube and gel, respectively. A CFA > 5, which is considered high risk for HDFN,^17^ was equivalent to titers of 128 and 512 for tube and gel, respectively.

### 3.2 Confounding factors

The full data set was analyzed for confounding factors. The mean gel to tube ratios were calculated for three subgroups within the cohort, including: samples that were processed fresh versus after one freeze/thaw cycle (*N* = 470); samples that contained additional red blood cell antibodies beyond anti‐D (*N* = 148; 7 samples contained historic antibody specificities with no antibody detected in the current sample and not used in the analysis); and samples from pregnant (*N* = 222) versus nonpregnant patients (*N* = 397; 16 patients with no obstetric status). Table 3 shows no significant differences within the first two subgroups; however, a significant difference was identified in the mean gel to tube ratios for samples obtained from pregnant versus nonpregnant patients (3.23 vs. 2.67; *p* < 0.001).

**TABLE 3:** Samples tested as potential confounding variables.

Subgroup	Log2 (gel/tube) mean (95% CI)		*p*‐value
	Yes	No	
Thawed sample (*N* = 470)	2.78 (2.56–2.99)	2.70 (2.44–2.96)	.55
Other antibodies present (*N* = 141)	2.89 (2.59–3.18)	2.72 (2.52–2.93)	.23
OB patients (*N* = 222)	3.27 (2.96–3.57)	2.66 (2.45–2.86)	<.0001

## 4 DISCUSSION

Antibody titration is a key tool for monitoring HDFN,^20^, ^21^ and for pregnant patients with anti‐D‐antibodies, a tube titer of ≥8 is often used as a trigger to begin monitoring for signs of fetal anemia. The tube method of titration testing is labor‐intensive and known for imprecision, while gel‐based technologies are more precise, more sensitive for detecting antibodies against the D‐antigen, and can be automated. Laboratories considering a change from tube to gel titration testing need to consider the shift in titer values caused by the more sensitive gel technology. This multicenter study measured D‐antibody titers in parallel using tube and gel methods. The results can help delineate the difference in titer levels and should help allay the doubts of clinicians who may be initially uncomfortable with the higher titer values generated by gel testing.

The greater sensitivity of gel versus tube in titrating anti‐D is illustrated by the 2.5‐ to 3‐fold increase in mean titers reported in this study and the finding that 155 samples that were negative in tube had titers of 1–64 in gel. Other studies comparing tube and gel for D‐antibody titers have reported 0‐ to 5‐fold differences in tube and gel titers,^8^ but an analysis of these studies revealed important inter‐study variability, including the use of reagent RBCs with different Rh system phenotypes (R_0_r, R_1_R_1_, R_1_R_2_). The current study used consistent lots of RBC reagent cells (R_2_R_2_) and standardized standard operating procedure (SOPs) across sites; this allowed low inter‐site variability. While R_2_R_2_ cells are not required for testing, they were selected because this phenotype has been associated with reduced variability in D‐antigen expression when compared to R_1_R_1_ and R_1_R_2_ RBCs. Also, R_2_R_2_ reagent red cells are readily available from commercial providers because of their strong antigen expression.^22^, ^23^

The observed increase in mean titers was consistent across lower tube dilutions, but decreased at titers ≥128 and were equivalent at a 1:1024 dilution (Figure 1). At higher titers, the inherent differences in sensitivity between the tube and gel methods become less significant, resulting in similar titer values. This observation may be explained by the study protocol, which called for 1024 as the final dilution to be tested. As a result, samples with the highest level of antibodies would yield the same result with tube and gel. Importantly, the clinically relevant titers that would trigger a higher level of care in pregnant patients (32–128) showed a similar difference between tube and gel titers.

Although the mean difference in titers was consistent at low and middle‐range titers, a wide range of tube to gel titer values was observed (see Table 2 and Figure 3). All sites reported a similarly wide range, which was likely due to the relative imprecision and wide variability associated with tube testing. The AABB Precision Comparison Study found that gel titration demonstrated superior precision and reduced variability compared to tube titration.^24^

CFA is the recommended method for anti‐D detection in the United Kingdom,^18^ but financial pressure has led to interest in other methods that could be used.^19^ This study contained a subset of samples originally tested by CFA in Wales and the Republic of Ireland and then shipped to a study site for parallel tube and gel titer testing. CFA values ≤0.4 IU/mL are associated with a low risk of HDFN,^19^ whereas CFA values >5 IU/mL have been validated as a threshold for clinically significant levels of anti‐D.^17^ In the present study, the comparison of CFA quantification of anti‐D versus tube and gel titer testing showed good concordance. Most importantly, nearly all samples with a CFA ≤ 0.4 IU/mL had tube titers less than 4. The mean gel titers were 16, which should be below the level of clinical significance, based on the results of this study. Samples with a CFA > 5 IU/mL were equivalent to titers ≥128 and 512, for tube and gel, respectively.

Potential study confounders were tested, but only the patient's pregnancy status showed a significant difference in mean titer values (Table 3). This may be due to sample bias, with more samples submitted for pregnant patients with higher titers. Additional studies will be needed to better understand this finding.

The strengths of this study include the large sample size and the use of consistent RBC reagents and testing protocols to provide results that could be generalized to labs using R_2_R_2_ reagent red cells.

The limitations of this study include the use of 1024 as the final dilution tested, rather than carrying each sample to its final titer. However, once titers achieved this level in gel, the paired titer in tube significantly exceeded the critical titer used for tube testing. Also, the study was confined to laboratory testing, with no attempt at clinical correlations between lab values and neonatal outcomes. There was no attempt to “send‐around” samples to characterize inter‐lab variations in testing. Importantly, this study used a single gel‐based method and a single manufacturer's automation system. It is unknown whether the results of this study are generalizable to other gel‐based systems. As a result, users of other gel‐based methods must perform their own internal validation to determine the difference in tube‐based testing versus another gel‐based method. Finally, the study tested only a limited number of CFA samples from two sites, which makes these results difficult to generalize.

In conclusion, this study stands as the largest and most rigorous comparison to date of tube and gel‐based D‐titration methods using patient samples. This research helps correlate critical tube titer values of 8–16 with gel titers of 32–128, providing guidance for clinicians transitioning to gel‐based methods. This study also shows a good correlation between CFA and tube/gel titers, which could help support a transition from CFA to titer‐based testing. Given the established imprecision of traditional tube testing, the greater sensitivity and consistency of gel testing confer an advantage when screening for anti‐D levels in pregnant patients. While this study offers evidence for the advantages of gel testing in the laboratory, further research is needed to establish a definitive clinical correlation between elevated D‐titer values obtained by gel methods and actual neonatal outcomes. This will ensure that the improved precision and sensitivity translate directly into better patient management and reduced HDFN‐related morbidity and mortality.

## FUNDING INFORMATION

QuidelOrtho provided financial support in the form of reagent red cells and shipping of samples.

## CONFLICT OF INTEREST STATEMENT

Claudia S Cohn received honoraria from QuidelOrtho.

## Data Availability

The data that support the findings of this study are available on request from the corresponding author. The data are not publicly available due to privacy or ethical restrictions.
